# Splenic Arterial Embolization in the Treatment of Severe Portal Hypertension Due to Pancreatic Diseases: The Primary Experience in 14 Patients

**DOI:** 10.1007/s00270-015-1199-8

**Published:** 2015-08-25

**Authors:** Qi Wang, Bin Xiong, ChuanSheng Zheng, Ming Liang, Ping Han

**Affiliations:** Department of Radiology, Union Hospital, Tongji Medical College, Huazhong University of Science and Technology, 1277 Jiefang Road, Wuhan, 430022 People’s Republic of China

**Keywords:** Clinical practice, Interventional oncology, Arterial intervention, Venous intervention

## Abstract

**Objective:**

This retrospective study reports our experience using splenic arterial particle embolization and coil embolization for the treatment of sinistral portal hypertension (SPH) in patients with and without gastric bleeding.

**Methods:**

From August 2009 to May 2012, 14 patients with SPH due to pancreatic disease were diagnosed and treated with splenic arterial embolization. Two different embolization strategies were applied; either combined distal splenic bed particle embolization and proximal splenic artery coil embolization in the same procedure for acute hemorrhage (1-step) or interval staged distal embolization and proximal embolization in the stable patient (2-step). The patients were clinically followed.

**Results:**

In 14 patients, splenic arterial embolization was successful. The one-step method was performed in three patients suffering from massive gastric bleeding, and the bleeding was relieved after embolization. The two-step method was used in 11 patients, who had chronic gastric variceal bleeding or gastric varices only. The gastric varices disappeared in the enhanced CT scan and the patients had no gastric bleeding during follow-up.

**Conclusions:**

Splenic arterial embolization, particularly the two-step method, proved feasible and effective for the treatment of SPH patients with gastric varices or gastric variceal bleeding.

## Introduction

Sinistral (left-sided) portal hypertension (SPH) is characterized by obstruction of the splenic vein leading to isolated gastric varices, mainly due to pancreatic diseases such as pancreatitis and pancreatic tumor [1–4]. The consequences of SPH are potentially fatal.

The direct cause of the occlusion is usually thrombosis in the splenic vein or compression by masses or cysts. Blood from the spleen flows towards the stomach via the short gastric veins and then flows towards the portal vein [2, 4]. Collateral circulation from the short gastric vein leads to the dilation of stomach submucosal vessels and subsequently increased blood flow and venous pressure, resulting in isolated gastric varices. These isolated gastric varices are the characteristic clinical feature of SPH, and may cause life-threatening gastric bleeding [2, 5]. The incidence of gastric bleeding in SPH varies from 4 to 72 % [4, 6].

Although the incidence of SPH has increased gradually in recent decades, there is no consensus on treatment strategy. In previous clinical studies, it was shown that splenectomy is an effective treatment for symptomatic or asymptomatic SPH. Yet, there is controversy regarding whether splenectomy is necessary for the prevention of gastrointestinal bleeding in asymptomatic patients [6–9]. Additionally, pancreatitis or the pancreatic tumor may activate inflammation and cellular adhesion, pancreatitis or pancreatic malignancy may make a splenectomy more technically challenging.

Partial splenic embolization with particle embolic agents has been well accepted for treating hypersplenism and portal hypertension caused by liver cirrhosis [10]. Moreover, total splenic arterial embolization with coils has been successfully applied in the treatment of splenic artery aneurysm [11]. In this retrospective study, we report our experiences using partial splenic embolization combined with splenic arterial embolization for the treatment of SPH patients with or without gastric bleeding.

## Methods

### Patients

Fourteen patients (12 men and 2 women; 22–59 years) with the presence of SPH underwent interventional therapy between August 2009 and May 2012, and were retrospectively analyzed in the present study. The causes of SPH were chronic pancreatitis in nine patients, acute pancreatitis in four patients, and pancreatic tumor in another one patients. Gastric bleeding occurred in eight patients who had melena, hematemesis, or both. Five of the eight patients had a chronic course with melena, and the other three patients had acute gastric bleeding with melena and hematemesis. Six asymptomatic patients were diagnosed during the follow-up of chronic pancreatitis.

Regarding clinical manifestations, ten patients had symptoms of postprandial fullness. Patients with hepatic cirrhosis-related portal hypertension were excluded. The ethics committee approved the study. All of the included patients provided signed informed consent.

### Diagnosis

The diagnosis of SPH was based on the results of CT, magnetic resonance imaging (MRI), clinical manifestation, endoscopy, and history. Enhanced CT/MRI provided detailed images of the liver, pancreas, and spleen, as well as the morphology of the abdominal vessels such as splenic arteries, splenic veins, portal veins, and collateral veins. The gastric varices were confirmed by the endoscopy. Patients with peptic ulcer were excluded from the current study.

**Fig. 1:**
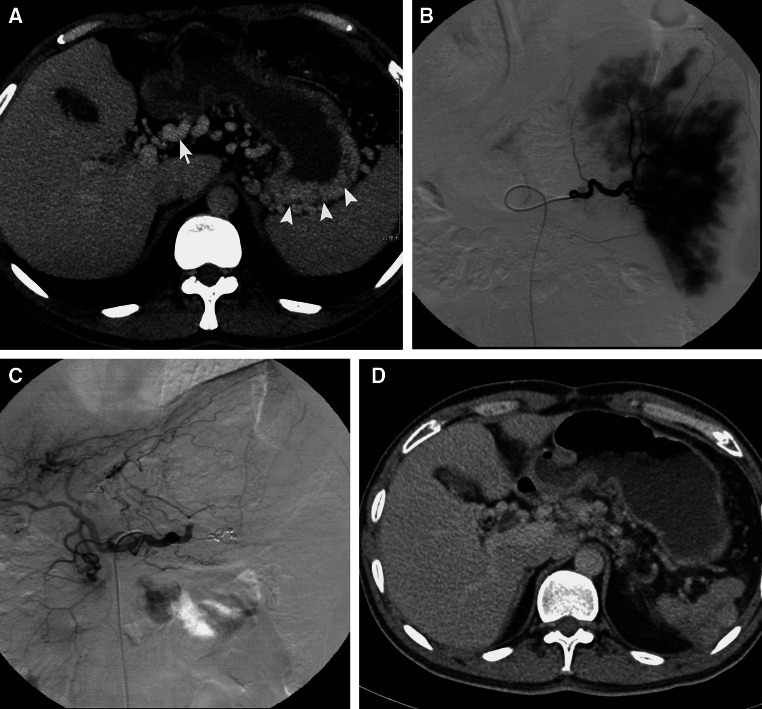
The spleen was embolized with the two-step technique in a representative patient with gastric variceal bleeding caused by chronic pancreatitis. **A** Collateral veins (*arrow heads*) in the stomach fundus drains from splenic vein and the coronary vein (*arrow*). **B** Angiogram of the splenic artery before the second embolic therapy showed the residual spleen stain. **C** Angiogram of the splenic artery after total embolization of splenic artery. **D** Atrophied spleen (*arrow*) 6 months after splenic arterial embolization

For the 14 included patients, the splenic vein vanished completely or partially in the CT/MRI images. Multiple collateral veins in the splenic hilum were detected in 14 patients. Blood from the spleen drained into the portal system via the following courses: (1) short gastric vein–gastric fundal vein–left gastric vein–splenic vein; (2) gastroepiploic vein–superior mesenteric vein; (3) short gastric vein–left gastric vein–splenic vein; (4) gastroepiploic vein–superior mesenteric vein; and (5) splenic vein–inferior mesenteric vein–superior mesenteric vein (Fig. 1). One of the three patients with an affected portal vein had cavernous transformation of the portal vein; the blood drained into the portal vein via the left gastric vein and the gastroepiploic vein. Splenomegaly was confirmed in 14 patients.

### Splenic and Splenic Arterial Embolization

Polyvinyl alcohol particles (PVA) (Cook Incorporated, USA) and coils (Cook Incorporated, USA) were used for the embolization. All the patients signed consent forms before the procedure. The patients with acute gastric variceal bleeding were treated with one-step embolization, in which the spleen and splenic artery were embolized simultaneously. Firstly, the spleen parenchyma was embolized with polyvinyl alcohol particles to decrease the blood flow to the splenic parenchyma and gastric varices. Then the small-sized coils were placed in the hilar splenic artery and the larger-sized coils were placed in the proximal splenic artery.

For the patients with gastric varices or chronic gastric variceal bleeding, the 2-step embolic method was applied. Firstly, partial splenic embolization was performed to achieve infarction of up to 60–70 % of the splenic volume according the angiography. Partial splenic embolization reduces the blood flow to the splenic parenchyma and gastric varices, and therefore the gastric bleeding can be relieved. The second embolic therapy was performed after a month with the aforementioned procedures.

Embolization was performed via femoral artery access. Celiac trunk angiography was performed with a 5-F Yashiro catheter to detect the splenic artery. Indirect portography was performed to show the images of the splenic vein, portal vein, and collateral veins. The catheter was placed in splenic artery near the hilum, and angiography was performed to show the splenic parenchyma and the splenic vein, portal vein, and collateral veins. A microcatheter was used in a patient with splenic pseudoaneurysm to pass through the stenosis of the splenic artery caused by the compression of the pseudoaneurysm. PVA particles (300–1000 µm) were used to embolize the splenic parenchyma. Coils were used to embolize the splenic artery.

## Results

### General Results of the Embolization

The embolization was successful in all of the 14 patients. The 1-step method was performed in three patients (two patients with acute pancreatitis and one patient with chronic pancreatitis). The patient with chronic pancreatitis had splenic arterial pseudoaneurysm, which was embolized with an isolated technique. Gastric bleeding eased after the embolization and further stopped during the hospitalization.

The 2-step method was successful in the remaining 11 patients (eight with chronic pancreatitis, one with pancreatic cancer, and two with acute pancreatitis). Six patients of the eight patients with chronic pancreatitis, all without a history of gastric bleeding, had gastric varices diagnosed by CT scan, and one of them had hypersplenism; no gastric bleeding was observed during the follow-up after the embolization. The other four patients who underwent the 2-step method had gastric bleeding prior to the procedure, and no gastric bleeding was found during the follow-up. The patient with pancreatic cancer only received the first step of the embolization, and was lost during the follow-up.

### Adverse Events and Complications

Post-embolization syndrome, including abdominal pain in the left upper quadrant area and fever, was the most frequent complications observed during the follow-up. Prophylactic antibiotics were used to prevent infections. Analgesic antipyretics such as diclofenac sodium suppository and tramadol were used to control fever and abdominal pain. Infection occurred in one patient after the 1-step embolization, perhaps caused by spleen necrosis as indicated by CT scan; the platelet count was significantly increased compared with the preoperative level in this patient. For three patients with elevated platelet count (>904 × 10^9^/L, even up to 1372 × 10^9^/L in a patient), aspirin was administrated to prevent thrombosis-related complications and disseminated intravascular coagulation. Constipation occurred in five patients and was relieved after the administration of laxatives. Severe complications such as splenic abscess and rupture of spleen did not occur in any of the patients.

### Follow-Up Outcomes

The patients with acute gastric variceal bleeding received enhanced CT scan in a month after embolization. Gastric varices and collateral circulations reduced obviously in the scan. In the second CT scan four months after the embolization, the gastric varices and collateral circulations had disappeared completely. During the follow-up, there was no recurrence of gastric bleeding.

For the patients treated with 2-step method, the gastric varices and collateral circulations reduced in the enhanced CT scan after the first embolization. The patients did not have gastric bleeding before the next embolization. After the second embolization, the gastric varices and collateral circulations disappeared gradually in the CT scans. There was no gastric hemorrhage during the follow-up. One patient with pancreatic cancer was lost during the follow-up.

## Discussion

In this retrospective study, we reported our experience implementing partial splenic embolization combined with splenic arterial embolization for SPH patients, in a 2-step protocol. Symptomatic patients with upper tract hemorrhage who received the procedure did not experience any relapse in gastric bleeding during the follow-up. Moreover, the gastric varices of all the patients disappeared gradually during the follow-up, as shown by CT scans. Procedure-related complications included fever and abdominal pain, which were tolerated by the patients. These results indicated that partial splenic embolization combined with splenic arterial embolization may be an effective and safe treatment strategy for patients with SPH.

Splenic vein occlusion caused by splenic vein thrombosis or compression from surrounding tissues has been recognized as the pathophysiological basis of SPH [2, 4, 5, 12]. Gastric variceal bleeding is one of the most serious complications of SPH and may be life-threatening [1, 13, 14]. Splenectomy has been proposed as treatment for SPH, because blood reflow to the varicose veins from the splenic vein is completely eliminated after removal of the spleen [8, 9, 15]. Normal circulation of the splenic vein can be reestablished with a stent implanted in the splenic vein through the thrombosis [16], but the patients should be selected strictly and most patients are not suitable for recanalization. Transjugular portosystemic shunt is not indicated for SPH because the shunt does not decrease the venous pressure in the gastric bundus. Splenic arterial embolization and partial splenic embolization have each been reported effective for the treatment of acute gastric bleeding due to SPH [14, 17, 18].

We developed splenic arterial embolization and partial splenic embolization to reduce variceal shunts around the stomach as much as possible. Partial splenic embolization is effective for the treatment of splenomegaly and hypersplenism, but these patients may suffer from serve postoperative reactions such as splenic abscess, sepsis, and rupture, due to the increased embolized spleen volume [10, 19–21]. According to published data and our clinical experience, the volume of spleen infarction should be controlled at 60–70 %. This not only relieves hypersplenism and gastric bleeding, but also decreases the incidence of serious complications. The splenic function returned after splenectomy for trauma and patients had low incidence of infection [22]. Splenic immune function was preserved in the patients after total or partial SAE for the blunt splenic injury [23]. The life-threatening infection in after SAE was relative rare with the covering use of prophylactic antibiotics. Routine vaccination was not indicted because the patients had normal splenic function after splenic artery embolization for trauma [24]. The embolic therapy discussed herein is minimally invasive, is of low risk, and is well tolerated by patients. In this study, the two embolic methods were applied according to the symptoms and signs of bleeding. Patients with acute gastric variceal bleeding were treated with the 1-step embolization, in which the spleen and splenic artery were embolized at the same session. For patients with chronic gastric variceal bleeding, or without a history of gastric bleeding but with gastric varices, the 2-step embolic method was performed, firstly, to achieve splenic infarction of up to 60–70 %. Splenic arterial embolization with coils decreases blood to the spleen parenchyma. Furthermore, this method decreases the risk of pseudoaneurysm [25]. The 2-step method is especially suitable for patients with splenomegaly and hypersplenism. Consistent with the literatures, abdominal pain and fever associated with post-embolization were the main clinical complications. These clinical manifestations may last for 3–10 days. For the patients treated with the 2-step method, less fever and abdominal pain may occur compared with those who receive the 1-step procedure. Prophylactic antibiotics and analgesic antipyretics may be applied. Severe complications, such as splenic abscess and rupture of the spleen, did not occur in these patients.

During the follow-up, the symptomatic patients did not have upper gastric bleeding after the procedure. However, it is controversial whether SPH patients without gastric bleeding require a splenectomy to cut off the blood flow to the gastric varices. Madsen et al. [4] in 1986 reported a high incidence of gastric bleeding (72 %) in SPH patients, and others reported similarly. Butler et al. [1] found that the incidence of splenic vein thrombosis caused by pancreatitis was 14.1 %, and the rate of gastric bleeding was 12.3 %. Sakorafas et al. [7] reported SPH diagnosed in 34 of 484 patients (7 %) with chronic pancreatitis. One of the asymptomatic patients with lateral pancreatojejunostomy and without splenectomy died due to gastric bleeding after three years. Heider et al. [6] in 2004 found that gastric bleeding occurred in 4 % of patients with gastric varices during the follow-up and splenectomy was needed in two patients. These data show that the incidence of gastric bleeding has decreased in SPH patients during the past decades, although the reasons are not clear.

It has to be mentioned that our study constitutes our primary experience of a limited number of cases, and more clinical evidence is needed. Moreover, in our study, the diagnosis of SPH was based on imaging technology including CT, magnetic resonance, and endoscopy. Recently, endoscopic ultrasound has been reported to be more sensitive in detecting paraesophageal varices and gastric varices than magnetic resonance or CT. Contrast-enhanced CT is also widely used in the diagnosis of splanchnic vein thrombosis. Future studies using these highly sensitive methods of examination during diagnosis and the follow-up of SPH patients are needed to confirm our results.

In conclusion, partial splenic embolization combined with splenic arterial embolization was an effective and safe treatment strategy for patients with SPH in this study.
